# Hippocampal Proliferation Is Increased in Presymptomatic Parkinson's Disease and due to Microglia

**DOI:** 10.1155/2014/959154

**Published:** 2014-08-14

**Authors:** Karlijn J. Doorn, Benjamin Drukarch, Anne-Marie van Dam, Paul J. Lucassen

**Affiliations:** ^1^Swammerdam Institute for Life Sciences (SILS), Center for Neuroscience, University of Amsterdam (UvA), Science Park 904, 1098 XH Amsterdam, The Netherlands; ^2^Neuroscience Campus Amsterdam (NCA), Free University (VU) Medical Center, Van der Boechorststraat 7, 1081 BT Amsterdam, The Netherlands

## Abstract

Besides dopamine-deficiency related motor symptoms, nonmotor symptoms, including cognitive changes occur in Parkinson's disease (PD) patients, that may relate to accumulation of *α*-synuclein in the hippocampus (HC). This brain region also contains stem cells that can proliferate. This is a well-regulated process that can, for example, be altered by neurodegenerative conditions. In contrast to proliferation in the substantia nigra and subventricular zone, little is known about the HC in PD. In addition, glial cells contribute to neurodegenerative processes and may proliferate in response to PD pathology. In the present study, we questioned whether microglial cells proliferate in the HC of established PD patients versus control subjects or incidental Lewy body disease (iLBD) cases as a prodromal state of PD. To this end, proliferation was assessed using the immunocytochemical marker minichromosome maintenance protein 2 (MCM2). Colocalization with Iba1 was performed to determine microglial proliferation. MCM2-positive cells were present in the HC of controls and were significantly increased in the presymptomatic iLBD cases, but not in established PD patients. Microglia represented the majority of the proliferating cells in the HC. This suggests an early microglial response to developing PD pathology in the HC and further indicates that neuroinflammatory processes play an important role in the development of PD pathology.

## 1. Introduction

Parkinson's disease (PD) is a progressive neurodegenerative disorder affecting 1-2% of the elderly population [1]. In addition to the well-known motor problems of PD patients, that are related to nigrostriatal dopamine deficits, also nonmotor symptoms are common. These symptoms strongly affect quality of life of PD patients as they include autonomic dysfunction, sleep problems, cognitive and neuropsychiatric changes [2–5], all changes unrelated to degeneration of the substantia nigra (SN). Apart from dopaminergic cell loss, the deposition of *α*-synuclein is a prominent neuropathological hallmark of PD. According to the staging concept described by Braak, *α*-synuclein deposition spreads over the brain in an anatomically predictable manner [6]. This coincides with the occurrence of nonmotor symptoms and can be observed already in early stages of PD, that is, in incidental Lewy body disease (iLBD) [7]. Moreover, *α*-synuclein can activate microglial cells, and neuroinflammatory responses are indeed important pathological features of PD [8–10].

**Figure 1:**
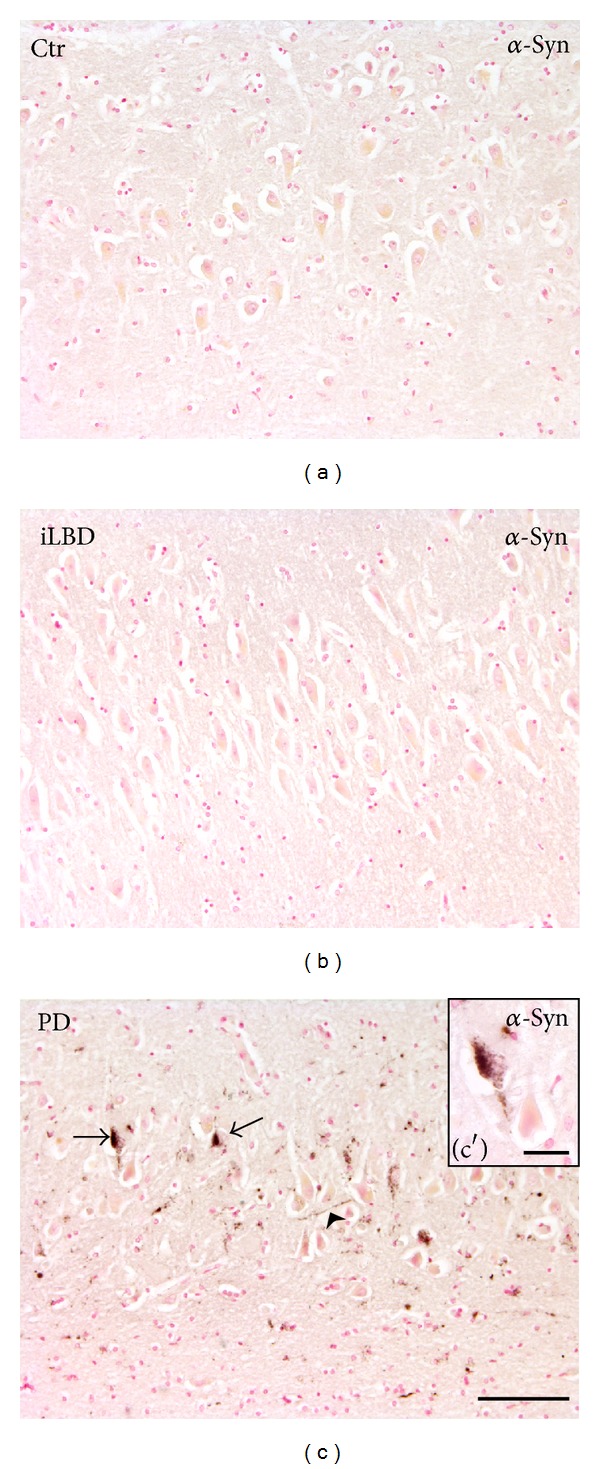
*α*-Synuclein pathology present in the hippocampus of PD cases. (a-b) *α*-Synuclein immunoreactivity (IR) in CA of control and iLBD subjects is absent compared to (c) *α*-synuclein IR (LBs: arrow, LNs: arrowhead) in the CA region of PD patients; bar (a–c) = 100 *μ*m; higher magnification (c′) bar = 20 *μ*m.

Accumulation of *α*-synuclein has been reported in the hippocampus (HC), which may contribute to the cognitive and depressive changes that represent prominent nonmotor symptoms in PD [11, 12] (Figure 1). Of interest, the HC is also one of the few brain regions where stem cells reside. In the hippocampal subgranular zone (SGZ), stem cells undergo proliferation before they migrate through the granular cell layer (GCL), where they eventually become newborn, functional neurons that contribute to network function. In addition to this unique process of adult neurogenesis, which is largely confined to the DG, stem cells in the HC can proliferate and respond to neurodegenerative conditions. For instance, overexpression of *α*-synuclein in PD models induces aberrant differentiation of neural progenitors and alters cellular plasticity in the hippocampus [13–15]. Also striatal deafferentiation, or the loss of dopaminergic neurons, affects both neurogenesis [16] and proliferation in the HC [17]. In addition, several Parkinson-related pathogens induce degeneration of human neural stem cells (NSCs) [18] or reduce neurogenesis [19]. Stimulation of cellular plasticity on the other hand, for example through exercise, antidepressant treatment or high frequency stimulation, reverses impairments in neurogenesis in PD models [20] and may even benefit PD patients [21, 22].

The discovery that the human brain contains stem cells has instigated extensive research into their proliferative responses during neurodegeneration and into their potential for brain repair. In related animal models for Alzheimer's disease (AD), changes in cellular plasticity and proliferation appear to depend on the extent of pathology and the severity of the disorder [23]. Regarding PD, stem cells and related cellular plasticity markers are also altered in relevant animal models [24–26], but only few studies exist on proliferative changes in human PD brain [26], most of which have focused on the SN [27, 28] and subventricular zone (SVZ) [29, 30]. In the human SN and SVZ, discrepant results have been obtained between different studies, and this topic remains subject of debate [26, 29, 31–34].

Thus far, little attention has been paid to cellular proliferative changes in the human HC, a brain region relevant for nonmotor symptoms in PD, like cognitive and depressive changes. Moreover, besides proliferation of NSCs, glial cells within the CNS may also proliferate in response to neuropathological changes. Indeed, in the AD hippocampus or in experimental multiple sclerosis, for example, proliferation of CNS resident microglia has been observed [35, 36]. Here, we first studied proliferative changes in prodromal and established cases of PD and, given the differential *α*-synuclein deposition, we include both the CA (nonneurogenic) and the DG (neurogenic) subregions. As activated microglial cells are present in the HC in PD and implicated in PD pathogenesis [9, 37], we also questioned whether microglial cells proliferate in these conditions. We selected hippocampal tissue of well-established and neuropathologically confirmed PD patients (Braak PD stages 4–6), matched control subjects (Braak PD stage 0), and incidental Lewy body disease (iLBD) cases (Braak PD stages 1–3) (Figure 1). As these latter cases lacked clinical symptoms of PD during their lives and did not receive dopaminergic medication, but displayed a neuropathological Braak *α*-synuclein deposition at autopsy [7], they can be considered a presymptomatic and prodromal state of PD. To assess cell proliferation, we used the well validated marker minichromosome maintenance protein 2 (MCM2) [38, 39] and colabeled it with the microglial marker Iba1 to investigate to what extent cell proliferation in the HC is accounted for by microglia.

## 2. Material and Methods

### 2.1. Postmortem Brain Tissue

Human postmortem hippocampal tissue was obtained from the Netherlands Brain Bank (NBB, Amsterdam, The Netherlands). In compliance with all local ethical and legal guidelines, informed consent for brain autopsy and the use of brain tissue and clinical information for scientific research was given by either the donor or the next of kin. Fourteen clinically diagnosed and neuropathologically verified PD patients (Braak PD stages 4–6) were selected as well as six clinically healthy controls without neurological or psychiatric disease, that displayed some PD *α*-synuclein pathology at autopsy (Braak PD stages 1–3) and were classified as iLBD cases. For the control group, nine healthy subjects without neurological or psychiatric disease and without any *α*-synuclein pathology (Braak PD stage 0) were included. The three groups were matched for gender and age; age of the control subjects ranged from 62 to 92 years, in the iLBD cases from 56 to 91, and in the PD patients from 59 to 96. All subjects were matched for postmortem delay and cerebrospinal fluid pH value. Donors who died of sepsis or stroke were excluded. Also Braak neurofibrillary tangles (NFT) and amyloid-beta (A*β*) plaques scores were matched between control subjects, iLBD cases, and PD patients, ruling out any possible difference in proliferation due to differences in AD pathology. The clinicopathological data of the patients and the Braak staging for PD and AD of all donors is summarized in Table 1.

Braak scores for AD and PD neuropathology were provided by the NBB and had been obtained after careful neuropathological evaluation of disease-relevant brain areas by experienced neuropathologists. The density and distribution of LBs/LNs, NFT, and A*β* plaques were determined using classic Bodian staining and immunohistochemistry for *α*-synuclein (Clone KM51, Novacastra, Bioconnect BV) and hyperphosphorylated tau (Clone AT8, Pierce, Rockford, IL) and A*β* (Clone 6F/3D, DAKO, DakoCytomation BV), respectively.

### 2.2. Tissue Processing

At autopsy, brain regions were dissected and immersion-fixed in 4% formaldehyde for four weeks, after which they were embedded in paraffin. From the paraffin blocks that contained the HC, 10-micrometer (*μ*m) sections were cut on a microtome, mounted, and dried in a stove overnight at 37°C before immunohistochemical analysis. Sections were mounted on positively charged glass slides (Menzel-Glaser SuperFrost Plus, Braunschweig, Germany).

### 2.3. Validation of MCM2 as Proliferation Marker in Human Brain Tissue

Various markers are available to identify cell proliferation or specific phases of the cell cycle in human postmortem brain tissue [39–42]. Of these, the minichromosome maintenance protein 2 (MCM2) is involved in the control of DNA replication and commonly used in cancer research as a reliable marker for detecting dividing and slowly cycling putative stem cells* in situ* [38, 43, 44]. MCM2 expression starts in early G1 and is maintained throughout the cell cycle, also in cells that proliferate without actually synthesizing DNA, and is thus present in higher numbers than, for example, the short-lived proliferation marker Ki-67 [38, 45, 46]. Moreover, the majority of the cells that express MCM2 coexpress the immature neuronal marker doublecortin [42] and MCM2 was therefore also used to study cellular plasticity in comparable tissues. Various tests to validate and confirm specificity of MCM2 and related markers have been performed by us and others before [38, 46, 47], for example, on samples of young rat brain and human colon, that were processed and embedded in the exact same way as the human brain tissue used in the current study [39, 41].

### 2.4. MCM2 Immunohistochemistry

Sections were heated in a stove for one hour at 56°C, before they were deparaffinized in xylene and rehydrated through a graded series of ethanol (100%, 96%, 90%, and 70%, resp.) and TBS. For subsequent antigen retrieval, sections were rinsed in 10 mM Tris buffer (pH 9.0) containing 1 mM EDTA (Tris-EDTA) and afterwards placed in preheated Tris-EDTA buffer in a steamer at 90–99°C for 30 minutes. After pretreatment, the sections were allowed to regain room temperature (RT), rinsed in Tris-buffered saline (TBS, pH 7.6), and incubated for 20 min in TBS containing 0.3% H_2_O_2_ and 0.1% sodium azide to block endogenous peroxidase activity. Nonspecific binding was blocked with 5% nonfat dried milk in TBS containing 0.5% Triton (TBS-T, pH 7.6; blocking solution) for 30 min at RT. Subsequently, sections were incubated overnight at 4°C with mouse anti-MCM2 (BM28, BD Transduction Lab 610700, mouse 1:600). Sections were then washed in TBS and incubated for 2 hr at RT in biotinylated goat anti-mouse IgGs (1:400; Jackson ImmunoResearch Laboratories Inc., West Grove, PA, USA) followed by HRP-labeled avidin-biotin complex (ABC complex, 1:400; Vector Laboratories, Burlingame, CA, USA) in TBS-T for 1 hr at RT. MCM2 staining was visualized using 3,3-diaminobenzidine (DAB, Sigma, St. Louis, USA) and counterstained with hematoxylin. After dehydration in graded ethanol solutions, sections were cleared in xylene and coverslipped in Entellan (Merck).

### 2.5. Immunofluorescence

For double-immunofluorescent labeling of microglial cells and MCM2 expression, Iba1 (WAKO chemicals, 019-19741, rabbit, 1:300) and MCM2 (1:1000) were used. Sections were pretreated with Tris-EDTA (pH 9.0) and primary antibodies were diluted in the blocking solution, as indicated above. After an overnight incubation at 4°C, the sections were washed and subsequently incubated for 2 h at RT with donkey anti-rabbit Alexa Fluor 488 labeled donkey anti-rabbit IgGs (Iba1) and biotinylated goat anti-mouse IgGs (MCM2) (1:400, Jackson ImmunoResearch, West Grove, PA, USA), followed by ABC complex (1:800) for 1 h and biotinylated tyramide enhancement in TBS for 20 min. Hereafter, sections were incubated with Alexa Fluor 594 labeled streptavidin (1:800, Jackson ImmunoResearch, West Grove, PA, USA). After washing, sections were coverslipped with Vectashield and examined using a confocal microscope (Leica TSC-SP2-AOBS; Leica Microsystems, Wetzlar, Germany).

### 2.6. Semiquantitative Analyses of MCM2-Positive Cells

**Figure 2:**
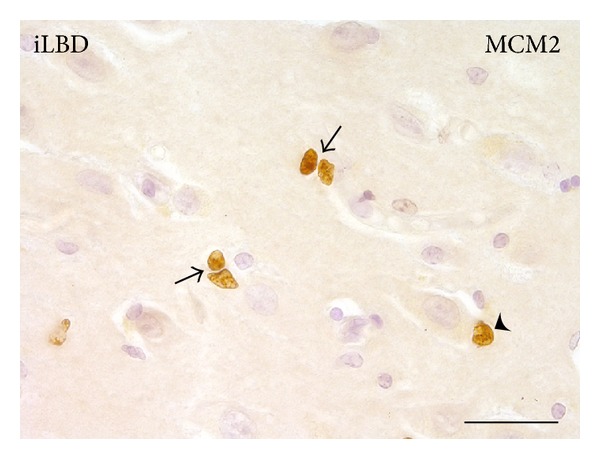
MCM2-positive cells in the hippocampal CA3 region of an iLBD case. High magnification of MCM2 IR reveals clear examples of doublets (arrows). The MCM2-positive cell indicated with the arrowhead is closely opposed to a large pyramidal neuron; bar = 25 *μ*m.

For standardization purposes, hippocampal sections were collected only around the anterior to midlevel of the HC of every subject and only when large dentate gyrus (DG) and Cornu Ammonis (CA) subregions were both present. Furthermore, MCM2-positive cells were included in the subsequent analysis only when they displayed a clearly immature, mitotic, and/or migratory morphology, and were located in an isolated manner (Figure 2). Semiquantification was performed by assessing the numbers of MCM2-positive cells present in the five main HC grey matter regions, that is DG, CA4, CA3, CA2, and CA1, at a 10x magnification and then expressed per surface area (region of interest, ROI, 1 mm^2^) using Cell^F^ Olympus Soft Imaging Solutions GmbH software (Tokyo, Japan).

### 2.7. Statistics

Statistical analyses were performed using the SPPS package version 20.0 (Statistical Product and Service Solutions, Chicago, IL, USA). The mean and standard deviation of the number of MCM2-positive cells were calculated for each group and within each subject for all the different subregions of the HC. The data did not meet criteria for normal distribution; thus statistical analyses were performed using nonparametric tests. Statistical analysis was executed with the nonparametric Kruskal-Wallis test to examine main group effects between controls, iLBD, and PD patients. Subsequently, Mann-Whitney *U* tests were performed as post hoc tests. Results were considered significant if they fell below an alpha of *P* ≤ 0.016, after Bonferroni correction. Within the same cases, statistical analyses for the different subregions of the HC were performed with the nonparametric related samples Friedman's analysis to examine main region effects, followed by paired Wilcoxon tests as post hoc tests. Results were considered significant if they fell below an alpha of *P* ≤ 0.02, family wise error- (FWE-) corrected. This critical value was established with SISA (http://www.quantitativeskills.com/sisa/calculations/bonhlp.htm), which uses the mean correlation between variables (*r* = 0.65) that are mutually correlated (i.e., number of MCM2-positive cells) for the alpha correction and allows one to perform a less stringent correction than the Bonferroni method for multiple comparisons.

## 3. Results

### 3.1. Increased Number of MCM2-Positive Cells in the HC of iLBD Cases

**Figure 3:**
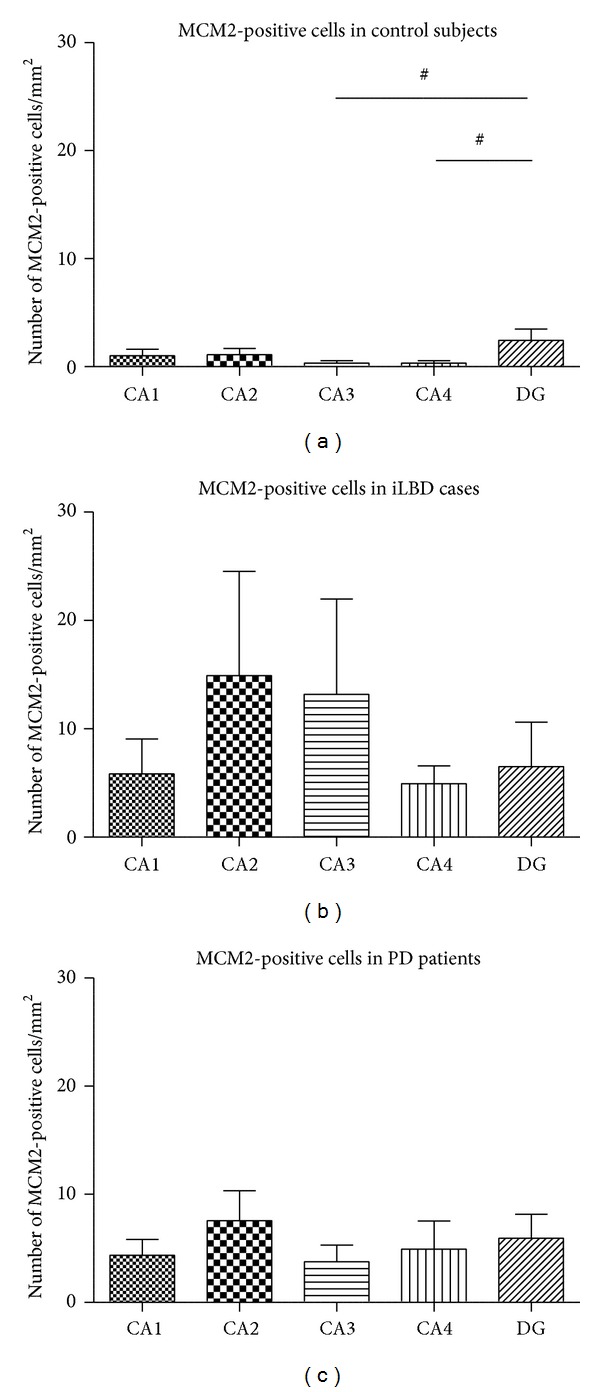
MCM2-positive cell numbers in control subjects, iLBD cases, and PD patients. Semiquantitative analysis revealed that in (a) control subjects, a significant main effect was present (*P* = 0.034; related nonparametric Friedman's analysis) with higher numbers of MCM2-positive cells in the DG compared to the hippocampal subregions (DG versus CA3 and CA4 ^#^ *P* = 0.043; DG versus CA1 *P* = 0.08 n.s.; DG versus CA2 *P* = 0.225 n.s.; nonparametric paired Wilcoxon post hoc test). (b, c) In the iLBD and PD cases, no differences were present between any of the subregions. Significance reached at alpha of *P* ≤ 0.02, FWE-corrected. Data represent mean ± SEM.

In the pyramidal layer and DG of the HC of all three patient groups, generally low numbers of MCM2-positive cells were found that displayed morphology typical for proliferating cells, such as a doublet shape and small size (Figure 2 and Figures 5(b), 5(e), and 5(h)). Except for a significant main effect in the number of MCM2-positive cells between the hippocampal subregions within the control group (Figure 3(a); *P* = 0.034, related samples Friedman's analysis), no significant differences were found between the subregions within each of the 2 different patient groups (Figures 3(b) and 3(c)). Further analysis revealed a trend towards an increase in the DG compared to the CA3, CA4, and CA1 within the control group (Figure 3(a); resp., *P* = 0.043, *P* = 0.043, and *P* = 0.08; nonparametric Mann-Whitney *U* test, significance reached at alpha of *P* ≤ 0.02, FWE-corrected; Ctr DG mean = 2.45 ± 1.03; Ctr CA3 mean = 0.31 ± 0.21; Ctr CA4 mean = 0.35 ± 0.25; Ctr CA1 mean = 0.93 ± 0.56).

**Figure 4:**
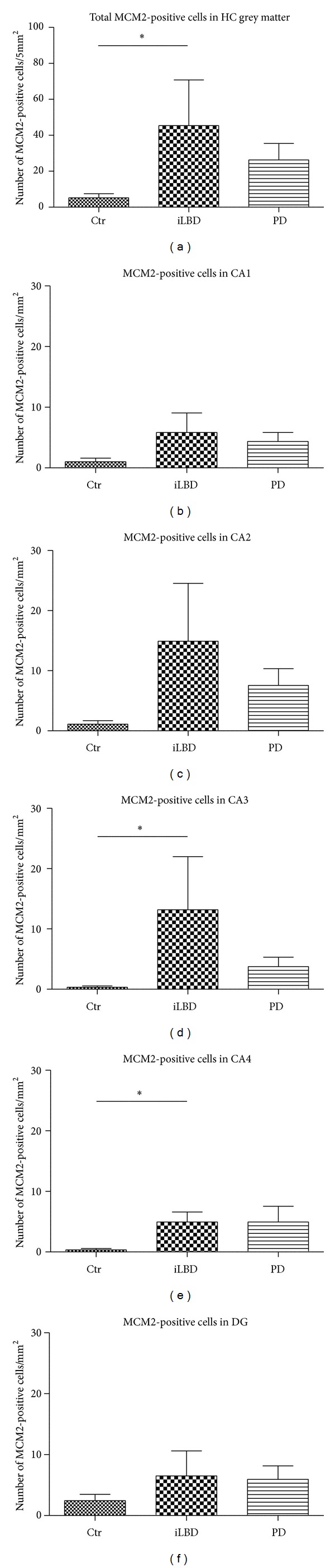
MCM2-positive cell numbers in the total HC and different hippocampal subregions. (a) The total number of MCM2-positive cells in the HC was significantly higher in iLBD cases compared to control subjects (*P = 0.004; nonparametric Mann-Whitney *U* test). (b–f) Comparing MCM2 IR per HC subregion between the 3 different groups revealed a significant increase in number of proliferating cells in hippocampal areas (d) CA3 and (e) CA4 (resp., *P = 0.001, *P = 0.003; nonparametric Mann-Whitney *U* test) of iLBD cases versus control subjects. Significance reached at alpha of *P* ≤ 0.016, Bonferroni-corrected. Data represent mean ± SEM.

Taking all data together revealed a significant increase in the number of MCM2-positive cells in the total hippocampal grey matter (CA1-CA4, DG combined) in the iLBD cases compared to control subjects (Figure 4(a); *P* = 0.004; nonparametric Mann-Whitney *U* test, significance reached at alpha *P* ≤ 0.016, Bonferroni-corrected; Ctr mean = 5.21 ± 2.23; iLBD mean = 45.24 ± 25.29; PD mean = 26.51 ± 9.25). When analyzing the subregions separately, a significant main group effect was found for CA3 and CA4 between control subjects, iLBD cases, and PD patients (resp., *P* = 0.006, *P* = 0.16; nonparametric independent samples Kruskal-Wallis analysis). Further analysis revealed there was a significant increase in the number of MCM2-positive cells in the CA3 and CA4 of the iLBD cases compared to the CA3 and CA4 of control subjects (Figures 4(d) and 4(e); resp., *P* = 0.001, *P* = 0.003; nonparametric Mann-Whitney *U* test, significance reached at alpha *P* ≤ 0.016, Bonferroni-corrected; Ctr CA3 mean = 0.31 ± 0.21; Ctr CA4 mean = 0.35 ± 0.25; iLBD CA3 mean = 13.05 ± 8.77; iLBD CA4 mean = 4.95 ± 1.67) and a trend towards an increase in the numbers of MCM2-positive cells in the other three hippocampal subregions of the iLBD cases compared to the control subjects (Figures 4(b), 4(c), and 4(f); CA1 *P* = 0.097; CA2 *P* = 0.082; DG *P* = 0.191; Ctr CA1 mean = 0.93 ± 0.56; Ctr CA2 mean = 1.17 ± 0.62; Ctr DG mean = 2.45 ± 1.03; iLBD CA1 mean = 5.88 ± 3.17; iLBD CA2 mean = 14.82 ± 9.58; iLBD DG mean = 6.45 ± 4.04). No significant differences were found between PD patients and control or iLBD cases in total HC or within any of the subregions (Figure 4).

### 3.2. Colocalization of MCM2 with Iba1 Microglial Cells in the HC

**Figure 5:**
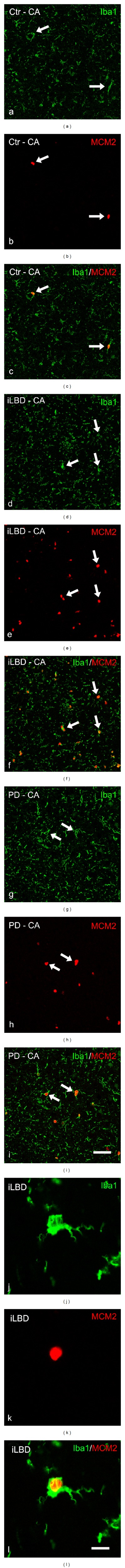
Colocalization of the majority of MCM2-positive cells with Iba1 in the hippocampal CA3 region of control subjects, iLBD cases and PD patients. (a–f) Representative confocal laser scanning microscopical images of double-immunofluorescent stained sections revealed that the majority of the MCM2-positive cells show colocalization with the microglial marker Iba1 (arrows in (c), (f), (i) and (j), and (k) and (l)). Iba1-positive microglia are depicted in green (a, d, and g) and MCM2 in red (b, e, and h). (j–l) Higher magnification of colocalization (l) between Iba1-positive microglia ((j), green) and MCM2 ((k), red); bar (a–i) = 40 *μ*m; (j–l) = 10 *μ*m.

**Figure 6:**
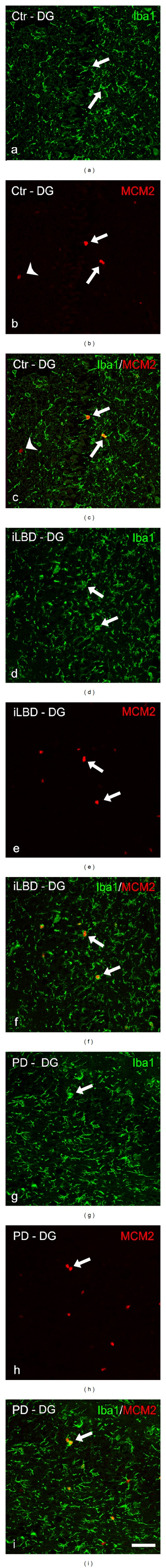
Colocalization of the majority of MCM2-positive cells with Iba1 in the hippocampal DG region of control subjects, iLBD cases, and PD patients. (a–f) Representative confocal laser scanning microscopical images of double-immunofluorescent stained sections revealed that the majority of the MCM2-positive cells show colocalization with the microglial marker Iba1 (arrows in (c), (f), and (i)). (b, d) Control subject shows one MCM2-positive cell with no colocalization with Iba1 (arrowhead). Iba1-positive microglia are depicted in green (a, d, and g) and MCM2 in red (b, e, and h); bar (a–i) = 40 *μ*m.

To determine whether microglia are proliferating, double immunofluorescence and confocal microscopical analysis revealed that the majority of MCM2-positive cells colocalized with Iba1-positive microglia (representative examples are shown in Figure 5). Of each patient group, 3 cases were double stained for Iba1 and MCM2 and the percentage coexpression was determined. In the control subjects, MCM2-positive cells were rare in the CA regions but always colocalized with Iba1 (100%). In iLBD cases, the highest number of MCM2-positive cells was present in the CA subregions. An average of 14 positive MCM2 cells were counted, of which an average of 13 were Iba1 positive, yielding 93% coexpression. In PD patients, fewer MCM2-positive cells were counted in CA regions compared to the iLBD group. An average of 6 positive MCM2 cells were counted, of which on average 5 were Iba1 positive, that is, 83%. In the DG, overall less MCM2-positive cells were counted; however, the percentages of colocalization with Iba1 were similar to CA regions. In general, quantification revealed >90% of the MCM2-positive cells in the HC to be microglia in control subjects, iLBD cases, and PD patients (representative examples are shown in Figures 5 and 6).

## 4. Discussion

We studied proliferating cells in the HC of control subjects, iLBD cases, and established PD patients. Using MCM2 as marker, no difference was observed in the amount of proliferating cells between control subjects and PD patients. However, in the presymptomatic iLBD cases, a clear increase in MCM2-positive cells was found. Interestingly, over 90% of the MCM2-positive cells were colabeled with Iba1, indicating that microglial cells are the main proliferating cells in the HC of iLBD cases and PD patients.

In control subjects, the low number of MCM2-positive proliferating cells observed in the HC is consistent with the low rates of hippocampal proliferation and neurogenesis found before in the aged rodent, primate, and human HC and in comparable PD brain tissue [30, 41, 48–51]. Within the control group, MCM2-positive cells were increased in the DG compared to other hippocampal regions (Figure 3(a)), which was of interest as most proliferation was beforehand expected to occur in this subregion. Interestingly, the differences between the DG and other subregions were no longer present in the iLBD and PD groups, with higher numbers of proliferating cells in all subregions (Figures 3(b) and 3(c)), suggesting that the disease process has triggered additional proliferative responses also outside the DG, and in fact throughout the HC.

When total counts in the combined HC were compared between the three cohorts, significant increases in proliferation were found in the iLBD cases, but no difference was present between the PD group relative to the control group (Figure 4(a)). This was unexpected as, based on experimental studies, lowered dopamine levels were expected to reduce hippocampal proliferation. Also the increase in iLBD cases was unexpected as no clinical symptoms were present in this cohort (yet) and although not quantified, their dopamine levels are assumed to be unaltered. However, there is also some DAT-SPECT data that reflects a decrease of striatal dopamine levels and nigral degeneration already in the premotor stage of PD [52–54]. Whether these cases can be readily compared to our iLBD cases remains to be answered. Subdividing the different HC subregions revealed that the significant increase in the entire HC in ILBD was mainly due to the CA3 and CA4 and to a lesser extent to the CA2 subregion (Figures 4(c), 4(d), and 4(e)). Since iLBD can be considered a presymptomatic state of PD, this result suggests that an early and disease-related induction of proliferation occurs in the HC.

Although earlier studies suggested that proliferation outside neurogenic regions may reflect “endangered” neurons that attempt to reenter the cell cycle [55], there are no indications that proliferation in the CA subregions will actually give rise to new neurons. Also the current morphology, consistent with a proliferative phenotype, and the localization of the MCM2 cells, that is, closely apposed to a pyramidal neuron and thus suggestive of a “satellite” cell (Figure 2, arrowhead), hinted that proliferation in nonneurogenic subregions likely reflects that of a nonneuronal cell type, for example, microglia. To confirm this, we used immunofluorescent colabeling of MCM2 with Iba1 and established that almost all proliferating cells in the PD HC represent microglia. The increase in iLBD cases relative to control subjects and PD patients suggests that proliferation of microglia occurs early in PD, prior to actual deposition of *α*-synuclein.

In most PD cases, neuronal populations in the HC show accumulation of *α*-synuclein [56, 57] which may activate the brain's immune system through microglia activation [58]. It is still debatable whether neuronal *α*-synuclein inclusions (Lewy bodies/Lewy neurites) cause microglial activation and/or neuronal death, or whether LBs/LNs act as protective “containers” and that it is the extracellular *α*-synuclein oligomers and fibrils that are toxic and activate microglia. On the other hand, neurons containing *α*-synuclein inclusions could communicate with microglia and activate them by neuron to glia signaling. Indeed, activation of microglia has been repeatedly shown in PD. While different microglial phenotypes are present in the SN, HC, and OB in PD, their functional role and hence the implications of microglia proliferation for PD etiology remain elusive [37, 59–61]. Similarly, the exact triggers for microglial proliferation are unknown. Transgenic mice overexpressing wild-type *α*-synuclein develop *α*-synuclein inclusions shortly after birth but show unchanged cell proliferation in their SVZ and HC at later ages [14, 62, 63]. In related prion disease models, proliferation of microglial cells is considered important in their turnover [64, 65] and to a larger extent to account for the expansion of the resident microglia population during, for example, prion disease development [66]. A related study on AD mouse models found changes in proliferation and microglia to coincide in time with increases in amyloid plaque load [67], suggesting that the accumulation of aberrant proteins like amyloid, and possibly also of *α*-synuclein, may trigger microglial proliferation.

While* in vitro *studies had already demonstrated that the aggregated form of *α*-synuclein can trigger microglial activation [10, 68], the HC of iLBD cases is largely devoid of *α*-synuclein depositions, and a role for soluble forms or *α*-synuclein oligomers can thus not be excluded. *α*-Synuclein oligomers can, for example, activate microglia via toll-like receptor-2 (TLR2) and thereby stimulate proinflammatory cytokine production [69, 70]. In agreement, in a separate study, we found TLR2 expression to be upregulated in microglia in the HC of iLBD cases [61].

In addition to increasing the cell population, an alternative, more speculative, interpretation of proliferation in microglia could be phagocytosis of *α*-synuclein. The release of *α*-synuclein and the subsequent uptake by neighboring neurons or glia suggest possibilities for cell-to-cell transfer and propagation that was recently proposed as conceptual model for proteinopathies [71, 72] and is consistent with the spatiotemporal progression of PD neuropathology over the brain [73]. Interestingly, neuron-to-glia transmission is accompanied by microglia activation [26, 74] and could thus underlie some of the proliferative changes observed in microglia in our current study. For instance, *α*-synuclein secreted by neurons is released into the extracellular space and can be taken up by microglia and phagocytosed. Several forms of *α*-synuclein have been found to induce microglial activation [10, 75, 76]. Similar to amyloid-beta, such secreted proteins can be “sensed” by microglia through toll-like receptors that could lead to the activation of inflammatory response genes and their proliferation [61, 69, 71, 77–79]. Another possibility is that proliferative changes reflect altered calcium signaling that was, for example, found to be dysregulated in microglia in close vicinity to amyloid plaques in AD brain [80]. These options are still speculative, and ongoing phagocytosis of *α*-synuclein inside microglia is technically difficult to visualize in postmortem human brain but may provide a possible explanation for the microglial proliferation we observed.

Our current hippocampal data are in contrast with a previous study on proliferation in the SVZ in PD and PD models [29], indicating that cell proliferation in the SVZ, and possibly also in the DG, may be under dopaminergic control [16, 29, 81, 82]. These authors used nestin and beta tubulin as markers and found significant reductions in proliferation in the HC of 3 PD patients and 5 PD patients with dementia, which they compared to 3 controls. Although different markers were used, we did not find any indication for a reduction in proliferation in the HC our cohorts, together comprising 20 patients and 9 controls. Another study on the SVZ failed to find differences in the proliferative capacity between controls and PD patients too [30]. While followed by an interesting debate [32, 33], this also suggested that proliferating progenitor cells are at least not reduced in PD. The methodological limitations that exist for visualization of neuronal proliferation in human postmortem brain [83–88] do not seem to hold explicitly for microglia proliferation. MCM2 can thus be used as a marker to determine also nonneuronal cell proliferation in human postmortem material.

In conclusion, the increase in numbers of proliferating cells in the HC of iLBD cases, a prodromal state of PD, but not in clinically established PD patients, indicates an early response to developing pathology in the PD HC. As almost all of the proliferating cells in the HC are microglia, this implies that neuroinflammatory processes may play an important role in ongoing PD pathology.

## Figures and Tables

**Table 1:** Clinical and neuropathological information of all included subjects.

C. number	Sex	Age	PMD (hrs.)	Braak staging		D	Region	Cause of death
				AD (NFT/A*β*)	PD (*α*-syn.)			
1	F	92	7:00	1A	0	NDC	HC	Acute death, pulmonary emboli
2	M	88	4:23	2A	0	NDC	HC	Gastrointestinal bleeding
3	F	84	6:55	1O	0	NDC	HC	Myelodysplasia
4	M	82	5:10	1O	0	NDC	HC	Unknown
5	M	62	7:20	1O	0	NDC	HC	Unknown
6	F	83	3:20	1B	0	NDC	HC	Legal Euthanasia
7	F	84	4:45	1O	0	NDC	HC	Heart failure
8	F	85	5:19	2B	0	NDC	HC	Natural death, pulmonary disease
9	M	78	<17:00	1O	0	NDC	HC	Heart failure

10	F	82	5:10	2O	2	iLBD	HC	Heart failure
11	M	86	4:00	2B	1	iLBD	HC	Respiratory insufficiency
12	M	56	5:00	0 (—)	2	iLBD	HC	Pneumonia
13	F	91	4:50	(—)	1	iLBD	HC	Exhaustion, colon carcinoma
14	M	84	7:20	1B	3	iLBD	HC	Prostate cancer
15	M	87	10:20	1A	1	iLBD	HC	Pneumonia, heart failure

16	M	83	4:50	1A	4	PDD	HC	Heart failure
17	F	59	9:35	1A	4	PD	HC	Shock due to blood loss in digestive tract
18	F	90	4:50	1B	4	PDD	HC	Unknown
19	F	70	7:05	2B	6	PDD	HC	Haematemesis by oesophagitis
20	M	84	9:00	1A	5	PDD	HC	Pneumonia and dehydration
21	F	87	5:25	2B	6	PDD	HC	Pneumonia
22	M	73	5:35	1A	5	PDD	HC	Direct cause unknown (morphine)
23	M	83	5:15	1B	6	PDD	HC	Pneumonia
24	F	84	7:25	2B	5	PD	HC	Old age, shortness of breath
25	F	96	7:10	1B	5	PD	HC	Old age
26	M	86	5:10	2B	5	PD	HC	Heart failure
27	M	71	5:50	1A	6	PD	HC	Respiratory failure
28	F	83	6:05	1O	4	PDD	HC	Cachexia by dementia, infarction
29	M	83	6:35	1B	6	PDD	HC	Pneumonia

D: clinical diagnosis; PMD: postmortem delay; NFT: neurofibrillary tangles; A*β*: amyloid-beta; *α*-syn: alpha-synuclein; NDC: nondemented control subject; iLBD: incidental Lewy Body disease; PD: Parkinson's disease; PDD: Parkinson's disease with dementia; HC: hippocampus.
